# Multisensory Integration of Naturalistic Speech and Gestures in Autistic Adults

**DOI:** 10.1002/aur.70042

**Published:** 2025-04-17

**Authors:** Magdalena Matyjek, Sotaro Kita, Mireia Torralba Cuello, Salvador Soto Faraco

**Affiliations:** ^1^ Humboldt‐Universität zu Berlin Berlin Germany; ^2^ Universitat Pompeu Fabra Barcelona Spain; ^3^ University College London London UK; ^4^ University of Warwick Warwick UK; ^5^ Serra Húnter Universitat Politècnica de Catalunya‐BarcelonaTech Barcelona Spain; ^6^ Institució Catalana de Recerca i Estudis Avançats (ICREA) Barcelona Spain

**Keywords:** audio‐visual speech, autism, EEG, iconic gestures, multisensory integration

## Abstract

Seeing the speaker often facilitates auditory speech comprehension through audio‐visual integration. This audio‐visual facilitation is stronger under challenging listening conditions, such as in real‐life social environments. Autism has been associated with atypicalities in integrating audio‐visual information, potentially underlying social difficulties in this population. The present study investigated multisensory integration (MSI) of audio‐visual speech information among autistic and neurotypical adults. Participants performed a speech‐in‐noise task in a realistic multispeaker social scenario with audio‐visual, auditory, or visual trials while their brain activity was recorded using EEG. The neurotypical group demonstrated a non‐linear audio‐visual effect in alpha oscillations, whereas the autistic group showed merely additive processing. Despite these differences in neural correlates, both groups achieved similar behavioral audio‐visual facilitation outcomes. These findings suggest that although autistic and neurotypical brains might process multisensory cues differently, they achieve comparable benefits from audio‐visual speech. These results contribute to the growing body of literature on MSI atypicalities in autism.

**Summary**

- When we listen to someone speak, seeing their face can make it easier to understand their words, especially in noisy environments.
- This study examined how autistic and non‐autistic adults process speech using both sound and visual cues, such as watching the speaker's mouth move.
- While both groups benefited equally from combining sound and visual information to understand speech, their brain activity patterns were different.
- Autistic adults relied on different brain processes than their neurotypical peers, suggesting that they might use alternative strategies to achieve similar outcomes.
- These findings highlight that differences in brain processing in autism do not necessarily mean difficulties, and they provide insight into how autistic individuals adapt to communicate in social settings.

## 1 Introduction

Face‐to‐face conversations rely on the processing and integration of auditory (acoustic, phonetic, and phonological cues) and visual information (lip movements, facial expressions, and body gestures). These cues collectively inform lexical access, syntax, semantics, and pragmatics (Brunellière et al. 2013; Brunellière and Soto‐Faraco 2015). In neurotypical^1^ populations, the integration of congruent visual and auditory information increases communication efficiency and speech perception, particularly in adverse listening conditions (Bernstein et al. 2004; Ross et al. 2007; Schepers et al. 2013; Sumby and Pollack 1954).

Compared to neurotypical development, autism is characterized by difficulties in social interactions/communication, repetitive behaviors, and sensory atypicalities (American Psychiatric Association 2013), with differences in the processing of single modality information as well as the integration of multisensory signals (Iarocci and McDonald 2006; Koelewijn et al. 2010; Marco et al. 2011; Stevenson, Segers, et al. 2014; Zhang et al. 2019), especially in the context of social cues, like speech (Stevenson, Siemann, et al. 2014). However, existing research has often used well‐controlled and idealized stimuli, far from the complexity of typical social contexts. Therefore, there remains a gap in understanding how multisensory integration (MSI) processes play out in more naturalistic social settings. Here, we address this gap by investigating behavioral and neural MSI in autistic^2^ and neurotypical adults using audio‐visual speech embedded in a naturalistic, linguistic, pragmatic, and social context.

Social interactions are not only dynamic and perceptually complex, but also multisensory in nature and unfold in parallel with other cognitive processes, like selective attention (Soto‐Faraco et al. 2019). To improve real‐world generalization, we prioritized ecological validity (Maguire 2012; Matusz et al. 2019) by including a rich socio‐linguistic context in our design: three persons playing a word game in an online video chat (this paradigm has previously been validated in Matyjek et al. (2024)). This setting provides natural context‐based pragmatics and semantic priors for meaningful speech stimuli, which are complete words (contrary to isolated syllables or out‐of‐context utterances; Stevenson, Siemann, et al. 2014; Williams et al. 2004). We also included a full view of the actors' torso and face because MSI benefit relies on multiple naturally produced *visual articulators* (Drijvers and Özyürek 2017), including orofacial movements, head, and eyebrow dynamics (Thomas and Jordan 2004; Yehia et al. 2002). Finally, in real‐life conversations, hand gestures are well coordinated with speech (Goldin‐Meadow and Wagner 2005; Kita and Özyürek 2003; McNeill 1992; Özyürek et al. 2007). *Iconic* gestures (representing characteristic actions or attributes; Kita and Özyürek 2003) are endowed with semantic information (Drijvers and Özyürek 2017). Integration of gestures with speech in neurotypical populations improves speech comprehension and shows characteristic neural effects (Drijvers and Özyürek 2017; Holler et al. 2014; Özyürek 2014), especially in adverse listening conditions (Drijvers and Özyürek 2017; Özyürek 2014; Drijvers, Vaitonytė, et al. 2019; Drijvers, van der Plas, et al. 2019; Holle et al. 2010).

Previous studies of audio‐visual and speech‐gesture integration in autism have focused mainly on children and adolescents, and their results are mixed. Some studies suggest atypicalities in single‐modality processing (Williams et al. 2004), while others point to specific differences in MSI (Stevenson, Siemann, et al. 2014; Silverman et al. 2010), some report atypicalities in both unisensory and MSI processing (Foxe et al. 2015; Irwin et al. 2011; Russo et al. 2010; Smith and Bennetto 2007; Stefanou et al. 2020; Stevenson et al. 2017), and some, based on behavioral outcomes, cannot clearly identify which process is responsible for group differences (Alcántara et al. 2004; Bebko et al. 2006). Despite possible atypicalities in early life, there is evidence for developmental improvement of MSI in autism (Foxe et al. 2015; Beker et al. 2018; Brandwein et al. 2013; Feldman et al. 2018), which may lead to typical integration performance—at least at a behavioral level—in adulthood (Keane et al. 2010; van der Smagt et al. 2007). In terms of speech‐gesture integration, one study (Silverman et al. 2010) found that while co‐speech gestures sped up gaze to targets for neurotypical adolescents, they delayed it for autistic peers, with no significant differences in task reaction times (RTs), making the gestural effect on autism unclear. However, more recent studies reported that autistic children (Dargue et al. 2021; Kurt 2011) and adults (Mazzini et al. 2023) benefit from co‐speech gestures in various tasks. For example, Mazzini et al. (2023) showed that the behavioral benefit from gestures in degraded speech is of comparable size in both autistics and neurotypicals. Together, given the mounting evidence for general MSI atypicalities in autism, particularly in childhood, semantically laden gestures seem to have the potential to facilitate otherwise atypical processing of multisensory information, especially in adulthood.

Importantly, variations in neural markers of MSI in autism (Stefanou et al. 2020) or in typical development (Santangelo et al. 2008) may not always correlate with behavioral changes, especially in adulthood. Thus, it is crucial to address both behavioral and neural correlates to understand MSI. Event‐related potentials (ERPs) are among the most common neural markers used to study MSI in speech processing. Multisensory stimuli modulate the amplitude of early ERPs, resulting in either enhanced or diminished activation compared to unisensory presentations (e.g., Besle et al. 2004) or the sum of both auditory and visual inputs (Molholm et al. 2002; Stevenson, Ghose, et al. 2014). However, ERPs can be challenging to use in naturalistic settings where the signal‐to‐noise ratio is low and precise time‐locking is difficult due to inherent differences in the onset of lexical, syntactic, and semantic processing across modalities (e.g., the precedence of gestures before speech onset; McNeill 2005). In a previous validation of the paradigm used in this study, we failed to detect reliable ERP components time locked to words, let alone distinguish between audio‐visual and unisensory conditions, despite clear MSI effects in oscillatory analyses in the alpha range (Matyjek et al. 2024). Hence, oscillatory EEG dynamics can index MSI (Varela et al. 2001; Senkowski et al. 2008) and are particularly useful with complex, ecologically valid stimuli (Matyjek et al. 2024). For instance, alpha/beta suppression and gamma power increase accompany gesture‐based enhanced speech comprehension (Drijvers et al. 2018a, 2018b), and alpha suppression can be interpreted as an indicator of heightened integration load (Matyjek et al. 2024).

In this study, we asked how multisensory speech is processed and integrated in autism under complex, naturalistic social conditions. We measured behavioral (accuracy of word recognition) and neural (alpha suppression) responses in autistic and non‐autistic adults, as they watched a video conference featuring multiple actors playing a word game, naming actions with corresponding gestures. Unisensory trials included either audio‐only (actor's camera off) or visual‐only (noisy audio) information, while multisensory trials included both. All trials included pink noise to create adverse listening conditions.

We expected that while the behavioral benefit from speech‐gesture MSI is similar in autistic (AUT) and neurotypical (NT) adults (Mazzini et al. 2023), MSI is realized differently at the neural level (linked to less suppressed alpha) in autism. Specifically, we predicted that: (1) The AUT group would show an audio‐visual benefit at a behavioral level, as measured with speech perception accuracy (higher accuracy in AV than in A and V); (2) The NT and AUT groups would show similar amounts of audio‐visual benefit in terms of behavior (here: similar AV − (A + V − A*V) difference in accuracy in AUT and in NT); (3) The AUT group would show an MSI effect for audio‐visual speech at the neural level, as measured with alpha suppression (stronger alpha suppression in the AV trials than in the sum of A and V trials); and (4) The AUT group would show a smaller MSI effect than NT at the neural level (less alpha power suppression). The hypotheses, predictions, and analysis pipelines were preregistered before data analysis (see https://osf.io/4765g).

## 2 Materials and Methods

### 2.1 Sample Size Calculation and Power Analysis

We performed a power analysis with the *pwr* package in R (Champely 2020), with a multiple regression power calculation with effect size *f* ^2^ = 0.205 (equivalent of *g* = 0.41 from a meta‐analysis of audio‐visual MSI in autism; Feldman et al. 2018), 80% power, alpha level of 0.05, and 2 numerator degrees of freedom (2 groups—1, times 3 conditions—1). This yielded a sample size of *N* = 25. However, we anticipated that our true effect size may be smaller given the naturalistic nature of our stimuli. Therefore, we planned to collect data from 35 participants per group, which would allow us to detect a medium‐large effect size of *f* ^2^ = 0.14.

### 2.2 Participants

Participants' characteristics are summarized in Table 1. All gave written consent to participate in the study. The study was approved by the Institutional Committee for Ethical Review of Projects at University Pompeu Fabra (CIREP‐UPF; No. 258). Participants were Spanish‐speaking volunteers between the ages of 18 and 60, with no intellectual disability and with normal or corrected‐to‐normal vision and audition.

**TABLE 1:** Demographic and trait characteristics of subject samples in all groups.

	AUT (*N* = 35)	NT (*N =* 35)	Group comparisons (AUT vs. NT)
Gender—F:M:O	16:14:5	16:19:0	ns
Sex—F:M:I	21:14:0	16:19:0	OR = 1.77
Age (years)	32.1 (9.6)	28.3 (10.3)	*t* = −1.59
AQ (total)	84.6 (10.3)	58.0 (10.7)	*t* = −10.09***
LSAS‐SR	84.1 (25.0)	37.7 (21.9)	*t* = −8.26***
Handedness—R:A:L	34:1:0	32:1:2	ns
IQ	113.1 (13.2)	111.4 (12.6)	*t =* −0.55
ADOS Total (*N* = 26)	6.8 (4.10)	—	—
ADOS Comm. (*N* = 25)	2.3 (1.4)	—	—
ADOS Social (*N* = 25)	6.1 (2.3)	—	—

*Note*: Count is provided for gender, sex, handedness, and means (with standard deviations) for all other items. F/M/O/I = female, male, other, intersexual; AQ = Autism Spectrum Quotient 10‐item Spanish version (Lugo‐Marín et al. 2019); LSAS‐SR = Liebowitz Social Anxiety Scale‐Self Reported; R/A/L = Right, Ambidextrous, Left; IQ = Intelligence Quotient measured with Raven's Progressive Matrices 2 (Raven and Raven 2003); OR = odds ratio in Fisher's Exact Test. Statistically significant tests were marked with ***for *p* < 0.001; ns = non‐significant.

AUT participants were recruited through invitations shared by collaborating autism centers in Barcelona, social media, flyers at public events, and word of mouth within the local autism community. All AUT participants had a confirmed diagnosis of autism spectrum disorder made in adulthood by professionals in specialized autism centers in Spain, in accordance with DSM‐5 criteria (American Psychiatric Association 2013). Of these, the diagnosis was confirmed by the Autism Diagnostic Observation Schedule (ADOS; Lord et al. 2000); in 26 participants and by the Autism Diagnostic Interview‐Revised (ADI‐R; Bölte and Poustka 2001; Lord et al. 1994); in 14 participants (additional diagnostic information in Supporting Information S1).

Using identical design and method, data from 39 NT participants were collected a few months earlier with the aim of comparing the groups, but were used first to validate the paradigm (Matyjek et al. 2024). Note that while these data were previously analyzed, the current study's hypotheses do not rely on those findings. Data from all AUT and NT were collected by the same experimenters, in the same location, with the same apparatus, and within 12 months. NT participants signed the same consent form as the autistic participants and underwent the same experimental procedure. To match the groups (AUT and NT) on count, biological sex, age, and IQ, we removed four NT data sets of the most extreme age and IQ scores (before analyzing the behavioral or EEG data), resulting in the final group of 35 NT participants, out of which none had a history of neuro/psychological/psychiatric disorders.

### 2.3 Task and Stimuli

**FIGURE 1:**
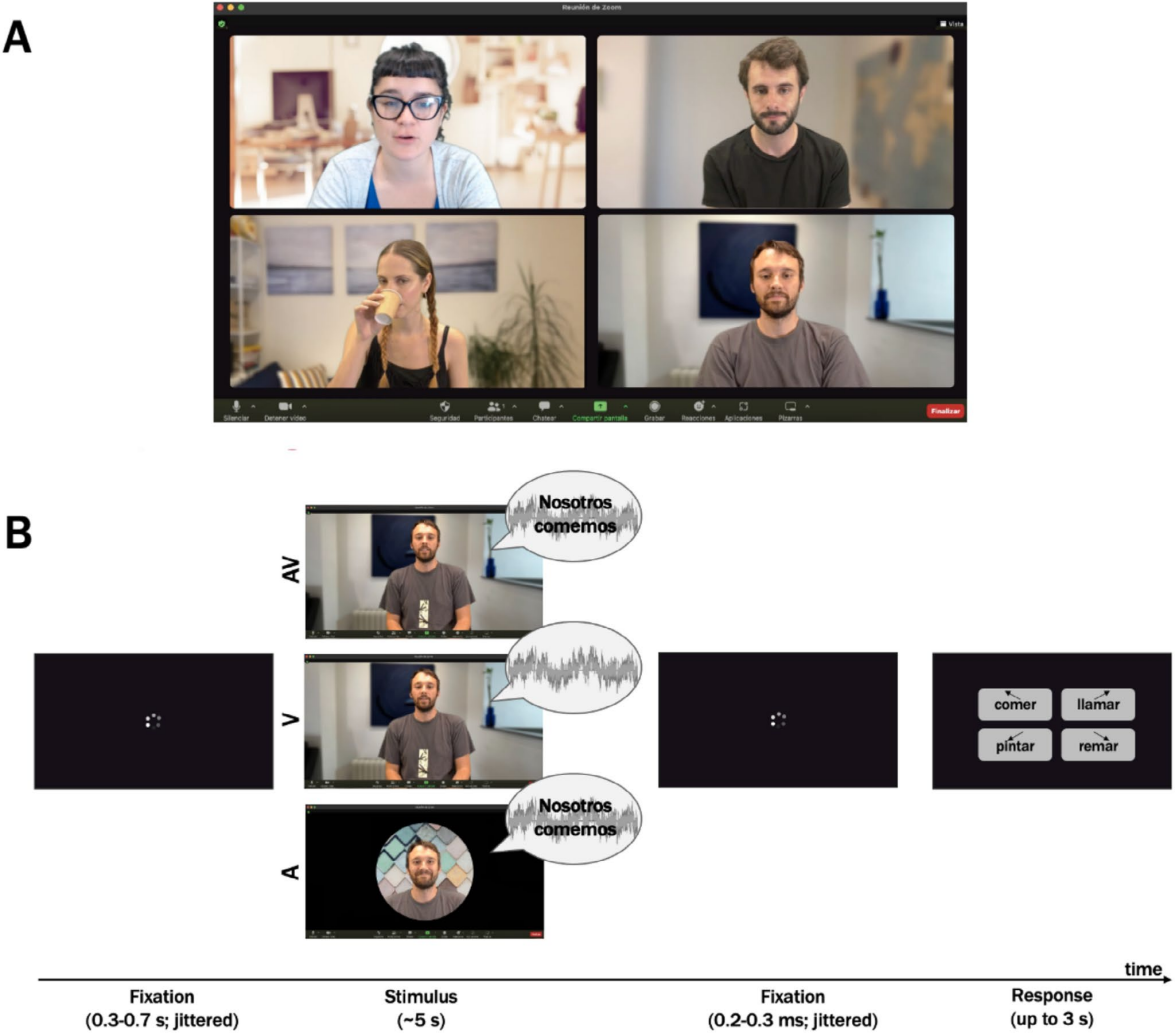
(A) Social situation: A videoconference with the teacher (top left) moderating a word game for three participants. (B) Schematic representation of the trial order. The gray waves in the speech bubble indicate pink noise to create an adverse listening condition. The time window of relevance was 0–1 s from the onset of the target word (in this example: “comemos”).

The task used was identical to that described in detail in Matyjek et al. (2024). In short, participants watched a (simulated) recording of a videoconference with three actors playing a word game (Figure 1; see a demo video of the paradigm at https://osf.io/kdj48). Actors uttered verbs matching a situation (e.g., “What do we do in a restaurant?”—“We drink.”) with corresponding gestures. Three conditions were introduced: audio‐visual (AV; actors both heard and seen), visual (V; actor's microphone “malfunctioning”), or auditory (A; camera “turned off”). Pink noise was added to each trial (audio speech‐to‐noise ratio = 0.44). Each of the 61 verbs was presented in all three conditions (183 trials). Videos were preceded (0.3–0.7 s) and followed (0.2–0.3 s) by a fixation symbol. Participants identified the verb by pressing one of four keys within 5 s, choosing between the correct word, a semantically similar word, a phonetically similar word, and an unrelated word (locations randomized).

Each target verb appeared once per condition and actor, with no more than three consecutive trials of the same modality (AV, A, and V). The word order was pseudo‐randomized using three versions with orthogonal condition assignments, balanced across participants. Training was repeated until instructions were clear. To ensure divided attention, participants were monitored for occasional visual or auditory glitches (“catch trials”; frozen frames or a burst of noise in the microphone, respectively). For a detailed design description, see Matyjek et al. (2024).

### 2.4 EEG Data Acquisition and Processing

The continuous EEG signal was acquired using the Brain Vision Recorder at a sampling rate of 500 Hz, with data collected from 61 active electrodes (actiCHAmp; Brain Products, Gmbh). Electrodes were positioned based on the extended 10–10 international electrode placement system and secured onto an elastic cap, with AFz serving as the ground. Impedances were maintained below 20 kΩ. To record eye movements, additional electrodes were positioned at the outer canthi of the right eye (HEOG) and below the left eye (VEOG). During recording, the online reference was set at the tip of the participant's nose. Offline pre‐processing of the EEG signal was conducted using Brain Vision Analyzer (Brain Products). First, we applied a 0.1 Hz high‐pass Butterworth filter (zero phase shift Order 8) to all data (no low‐pass filter was applied). Visual inspection was used to detect channels with poor signal quality (those that showed a flat signal (detached electrode) or that presented fluctuations in the voltage (standard deviation) larger than the rest of the electrodes of the set). Those were interpolated based on the neighboring channels using spherical splines of Order 4 (2.08% of all channels). Subsequently, average reference was performed. Continuous data were divided into segments from −1000 ms before to 3000 ms after the acoustic onset of the target verb. Local DC detrending was applied to segments to remove slow drifts in the baseline, and those containing large muscular artifacts were removed using a semi‐automatic procedure. An independent component analysis algorithm (restricted fast ICA) was used to identify and correct activity related to blinks and eye movements, resulting in an average removal of 2.81 components per subject. Manual artifact rejection was then performed on the remaining segments (which were similar in count and signal‐to‐noise ratio across groups and conditions; see Supporting Information S1). Finally, segments were divided into AV, A, and V trials for subject‐level and grand averaging. The processed data were exported to Matlab for further processing using the FieldTrip toolbox (Oostenveld et al. 2011).

### 2.5 Data Analysis

All statistical analyses were conducted in R v.4.3.2 (R Core Team 2023). The alpha level was set to 0.05. For all models, treatment contrasts were used with AV set as the reference level. Statistical assumptions were checked and met for each analysis; diagnostic plots can be found in the shared code and the accompanying html file (https://osf.io/2b6s3/). Note that Predictions 1 and 3 pertain to AUT data only, as the data, including all NT participants in this study, were already analyzed and reported elsewhere (Matyjek et al. 2024). Nevertheless, we also report the NT data here for completeness.

#### 2.5.1 Accuracy

For analyzing word recognition accuracy in AUT (Prediction 1), responses were correct if the participant chose the word matching the actor's utterance. Incorrect responses included choosing any of the other three words or failing to respond within 5 s. A generalized linear mixed model (GLMM) was used with a binary outcome variable (correct = 1 and incorrect = 0), condition (AV, A, and V) as predictors, and random intercepts and slopes for conditions within each subject and each verb. Further, we calculated MSI effects in the accuracy data, that is, whether the observed word detection probability in multisensory trials exceeds detection probability under multisensory stimulation predicted from unisensory stimulation. To do that, we used a probability summation equation (Stevenson, Ghose, et al. 2014): AV vs. (+‐A*), where AV, A, and V are the detection probabilities of correct responses in the corresponding conditions.

For analyzing the behavioral MSI benefit (to test Prediction 3), we calculated the absolute difference in detection rates between the AV and the probability summation of the unisensory conditions, that is, AV − (+‐A*). Then, these behavioral MSI benefit scores were compared between the groups with a two‐sided *t*‐test. While the preregistered methods for this analysis used a maximum unisensory equation (AV − max(A,V)), this approach does not adequately address statistical facilitation in the context of MSI (Stevenson, Ghose, et al. 2014). To better account for this, we report results using the probability summation equation (AV − (A + V − A*V)), which is more suitable for capturing the integration of multimodal signals. However, for transparency, all calculations based on the preregistered maximum unisensory approach are available in Supporting Information S1, showing a consistent pattern of results with the probability summation method.

#### 2.5.2 RTs

RTs may be less sensitive to MSI effects in our design because of the delayed response prompt introduced to avoid motor contamination in the EEG signal. For this reason, we preregistered an exploratory approach for the RT analysis. RTs faster than 200 ms and outliers (RTs outside the range median ± 2 × median absolute deviation; Leys et al. 2013) were removed. Additionally, RTs corresponding to trials in which the EEG data were rejected due to the presence of artifacts were also removed. Together, these exclusion criteria resulted in the exclusion of 9.96% of trials. We built a GLMM with condition, group, and their interaction as predictors. For pairwise comparisons within the model, Holm correction was administered via the *glht* function from the *multcom* R package (Hothorn et al. 2008). To estimate the main effects of the multilevel predictor condition, a Type II analysis of variance (ANOVA) was performed on the model. Because participants did not receive feedback for their performance, this model was built with all the available trials, including correct and incorrect ones. Nevertheless, we also built a model with correct trials only, which yielded very similar results (see section 7 in Supporting Information S1). To test for MSI non‐linearities, we calculated Miller's Race Model Inequality (Miller 1982) as described in Sinnett et al. (2008) as a benchmark.

#### 2.5.3 Alpha Suppression

As preregistered, we expected to observe centro‐parietal alpha suppression (frequency of interest; FOI: 8–13 Hz) in the first second (time of interest; TOI) after the onset of the target word. To identify the region of interest (ROI) for the analysis, we followed the method of Senkowski et al. (2007) described below and calculated the time‐resolved oscillatory power for each subject every 0.02 s using a Short Time Fourier Transformation (frequencies of interest: 5–30 Hz, in steps of 1 Hz, in segments of 0.5 s, Hanning window) for the interval of −1 to 2 s in Fieldtrip. Then, the time‐frequency was averaged across subjects for each condition. The ROI was defined based on visual inspection for all the subjects pooled together: CP1, CP3, P1, P3, and P5.

We expected that alpha suppression in the AV condition would be larger than in the probabilistic sum of suppression in single modality A and V trials. Because linear operations, like AV − (A + V) commonly used for ERP contrasts, are not appropriate for power responses computed with fast Fourier or wavelet transformations, we followed the analysis pipeline suggested by Senkowski et al. (2007) and previously validated for complex stimuli by our group (Matyjek et al. 2024). First, we combined the amplitude of A‐only and V‐only trials linearly to create A + V trials. Then, we applied a Morlet wavelet transformation (with seven cycles) to both multisensory (AV) and summed unisensory (A + V) epochs, converting them to the frequency domain. Then, the data were exported to R for further processing and analysis. Each epoch underwent absolute baseline correction relative to the mean value of the −300 to 0 ms period preceding the acoustic onset of the target word. Power values were then averaged across the ROI. Then, we performed bootstrapping on the A + V pairs, repeating the process 10,000 times to calculate their mean (in order to make sure the number of bootstrapping iterations does not inflate false alarm rate (FAR), we performed simulations that showed that FAR stabilizes above 50 iterations). Finally, to test Prediction 3 (that AUT shows an interaction MSI effect in alpha suppression), we calculated *z*‐scores of alpha power per participant ($\frac{\text{mean}\left(\mathrm{AV}\right)-\text{mean}\left(A+V\right)}{\mathrm{sd}\left(A+V\right)}$), and tested whether they significantly deviated from 0 (with a one‐sample *t*‐test, as we expected a suppression effect). As preregistered, we removed outliers defined as *z*‐scores over or below the median ± 2 × median absolute deviation (2 AUT and 4 NT).

To test whether AUT shows a smaller neural MSI effect than NT (Prediction 4), we tested the difference between the *z*‐scores in AUT and NT with a one‐sided *t*‐test.

#### 2.5.4 Exploratory Analysis: Spatio‐Temporal Unfolding of MSI Effects

Due to limited literature on interaction MSI effects in the frequency domain, this article also explores potential MSI effects beyond our planned alpha power analysis. We extended the MSI analysis pipeline (described above for alpha) to spectral bands theta (4–7 Hz), alpha (8–13 Hz), beta (14–30 Hz), and gamma (30–48 Hz) across time windows. For theta and alpha, we used 0–0.5 and 0.5–1 s windows; for beta and gamma, we divided the 1.5 s window into three 300 ms bins (0–0.3, 0.3–0.6, and 0.6–0.9 s). Each time window was absolute baseline‐corrected using the mean amplitude value −0.3 to 0 pre‐stimulus window. Wavelet transformations used 5, 7, 8, and 10 cycles for theta, alpha, beta, and gamma, respectively (see Matyjek et al. (2024) for validation with empirical and synthetic data).

## 3 Results

### 3.1 Behavior: Multisensory Gain in Accuracy

**FIGURE 2:**
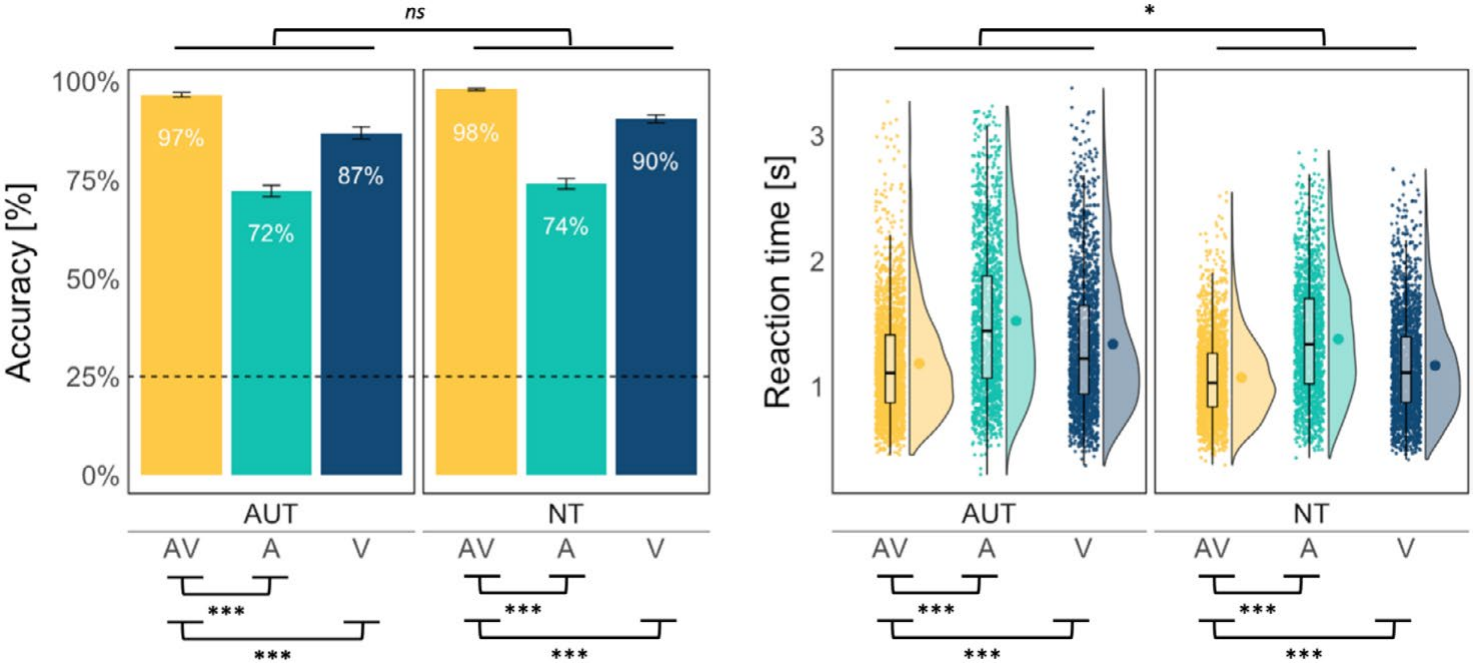
Mean accuracy (left panel) and reaction times (right panel) per group and condition. The error bars reflect standard error. The dashed line for accuracy marks the 25% chance of performance in the task.

A logistic regression revealed a significant main effect of condition, *X* ^2^ (2) = 78.79, *p* = 0.001 (see Figure 2, left panel). The odds of responding correctly in A trials were 94% smaller than in AV trials (1 − 0.06, *p* = 0.001, 95% CI = [0.04, 0.10]) and 78% smaller in V than in AV trials (1 − 0.22, *p* = 0.001, 95% CI = [0.14, 0.35]). Thus, Prediction 1 was supported: AUT showed a behavioral effect of multisensory over unisensory speech. A model including the factor group (AUT, NT) showed a similar pattern of results: better accuracy in AV than A or V conditions, with no group differences or interaction of group and condition (see Figure 2, left panel, and Supporting Information S1).

### 3.2 Behavior: AUT vs. NT Differences Audio‐Visual Gain

**FIGURE 3:**
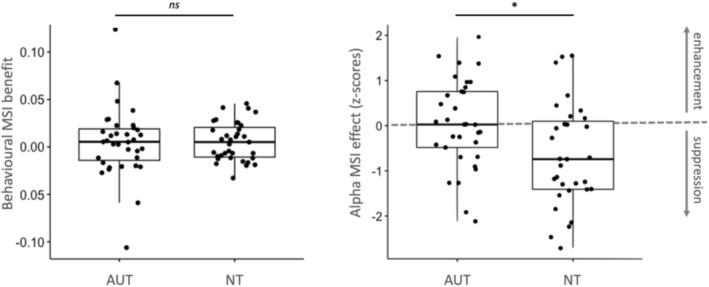
Behavioral benefits in word recognition (left panel) and neural MSI effect (*z*‐scores) (right panel) are found across the groups.

First, we tested the interaction MSI effects in the accuracy data. In the AUT and NT groups, 21 and 19 participants showed MSI effects (i.e., detection probability in multisensory trials was higher than the probability summation for the unisensory trials), respectively. These distributions did not differ significantly between the groups, *X* ^2^ (1) = 0.06, *p* = 0.81, BF01 = 3.05, *ϕ* = 0.03 (small effect). We compared the behavioral benefit (estimated as the difference in accuracy between AV and the probability summation of A and V) between AUT (mean = 0.005) and NT (mean = 0.006) with a *t*‐test, which yielded no significant effect, *t*(52.94) = −0.11, *p* = 0.91 (see Figure 3, left panel). We additionally compared a model including the group factor against an intercept‐only model, which yielded BF01 = 4.05, suggesting some evidence in favor of the intercept‐only model.

### 3.3 Behavior: Exploratory Analysis of RTs

An ANOVA on the GLMM with the factors condition (AV, A, and V) and group (AUT, NT) yielded the main effects of condition *X* ^2^ (2) = 1084.21, *p* < 0.001, and group *X* ^2^ (1) = 5.51, *p* = 0.02. The interaction also reached significance, *X* ^2^ (2) = 6.48, *p* = 0.04, but no pairwise comparisons of RTs between the groups survived correction for multiple comparisons, and BF showed strong evidence in favor of a model without the interaction term, suggesting that this effect was negligible (also see section 7 in Supporting Information S1 for this model with correct‐only trials, yielding similar results). Thus, RTs were faster for AV than V and for V than A, and this pattern held for both groups, although overall responses were faster in the NT than the AUT group (see Figure 2, right panel). The distribution of observed RTs in AV trials never surpassed the theoretical race model with RTs in A and V trials in either group (see Supporting Information S1 for details).

### 3.4 Neural Correlates: MSI Effects on Alpha Suppression

**FIGURE 4:**
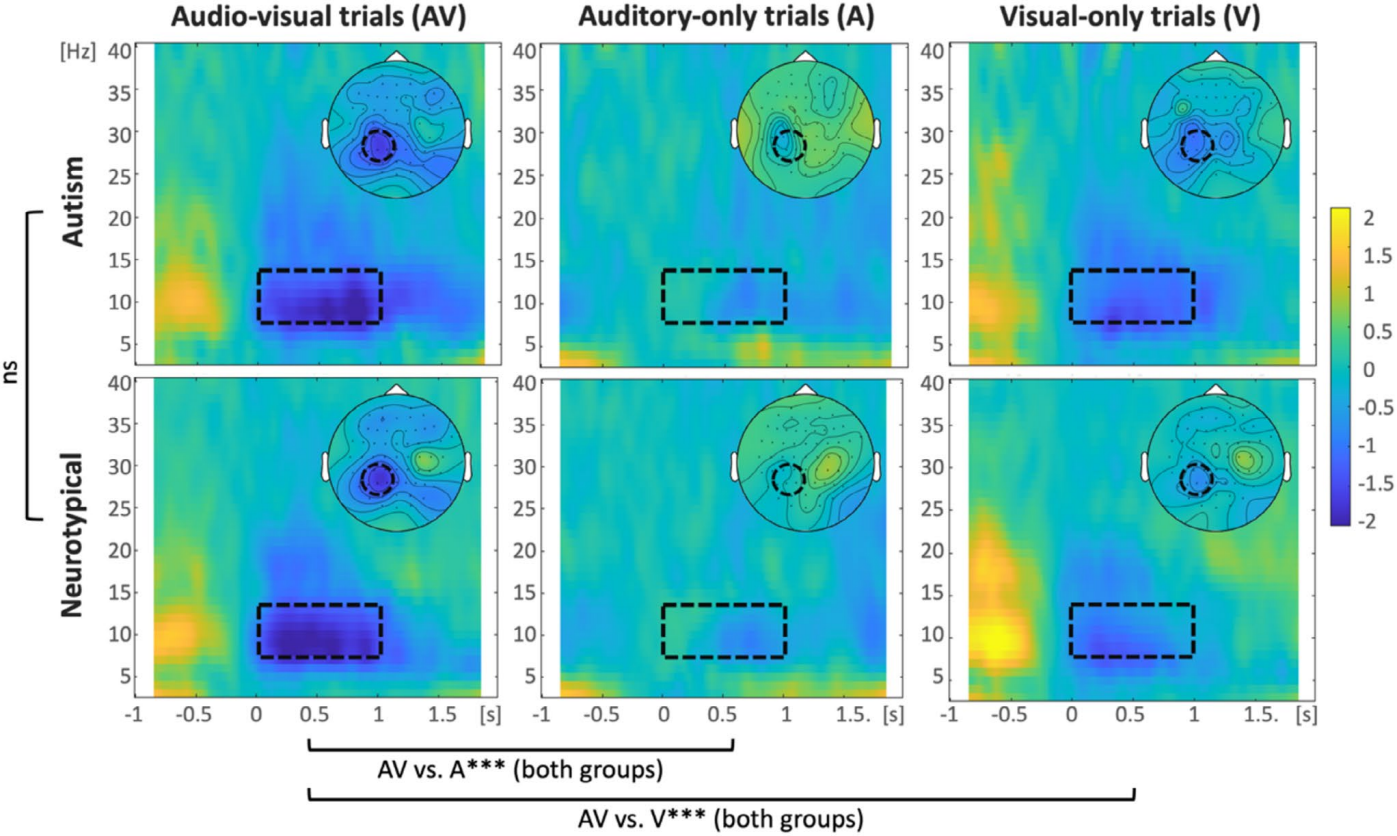
Time‐frequency representations (TFRs) per condition and group. Time in seconds is plotted on the horizontal axis, and frequency in Hz on the vertical axis. The dashed rectangles mark the time of interest (TOI; 0–1 s) and frequency of interest (FOI; alpha; 8–13 Hz). The TFRs are plotted for the region of interest (ROI) in the alpha analysis (CP1, CP3, P1, P3, and P5). The heads show the topography of the selected TOI and FOI, and the dashed circles mark the electrodes in ROI. The main effect of group (AUT vs. NT) and multi‐ vs. unisensory comparisons (AV vs. A, AV vs. V) are represented.

In the alpha suppression analysis, we first checked for simple audio‐visual benefits (AV vs. A and AV vs. V) in AUT. A linear mixed model revealed a main effect of condition, *F*(2,6083.5) = 9.43, *p* < 0.001, with stronger alpha suppression in AV compared to either A (est = 117.93, *t*(6083.4) = 4.31, *p* < 0.001) or V (est = 71.61, *t*(6083.6) = 2.62, *p* < 0.001). In a model including the factor group, the condition was still significant, *F*(2,12271.1) = 20.71, *p* < 0.001, but neither group nor the condition × group interaction was statistically significant (both *p*s ≥ 0.48; see Supporting Information S1). See TFRs per condition and group in Figure 4.

To test for potential interaction MSI effects in alpha suppression, we used *z*‐scores of the deviation between observed mean alpha power in AV trials from the mean of the probabilistic sum of alpha power in A and V trials, with the contrast AV ≠ A + V. Contrary to Prediction 3, we found no evidence for MSI effects in AUT, *t*(32) = 0.29, *p* = 0.62, *d* = 0.05 (small effect). On the other hand, NT showed a significant effect, *t*(30)= − 3.20, *p* = 0.002, *d* = 0.58 (medium effect).

### 3.5 Neural Correlates: MSI Effect Difference Between Groups

To test whether AUT showed a smaller alpha suppression MSI effect than NT (Prediction 4), we tested the difference between the *z*‐scores in AUT and NT with a one‐sided *t*‐test, which yielded significantly more negative *z*‐scores (i.e., stronger suppression) in NT than in AUT, *t*(59.19) = 2.65, *p* = 0.01 (see Figure 3, right panel).

### 3.6 Exploratory Analysis: Temporal and Spatial Unfolding of Neural MSI Effects

**FIGURE 5:**
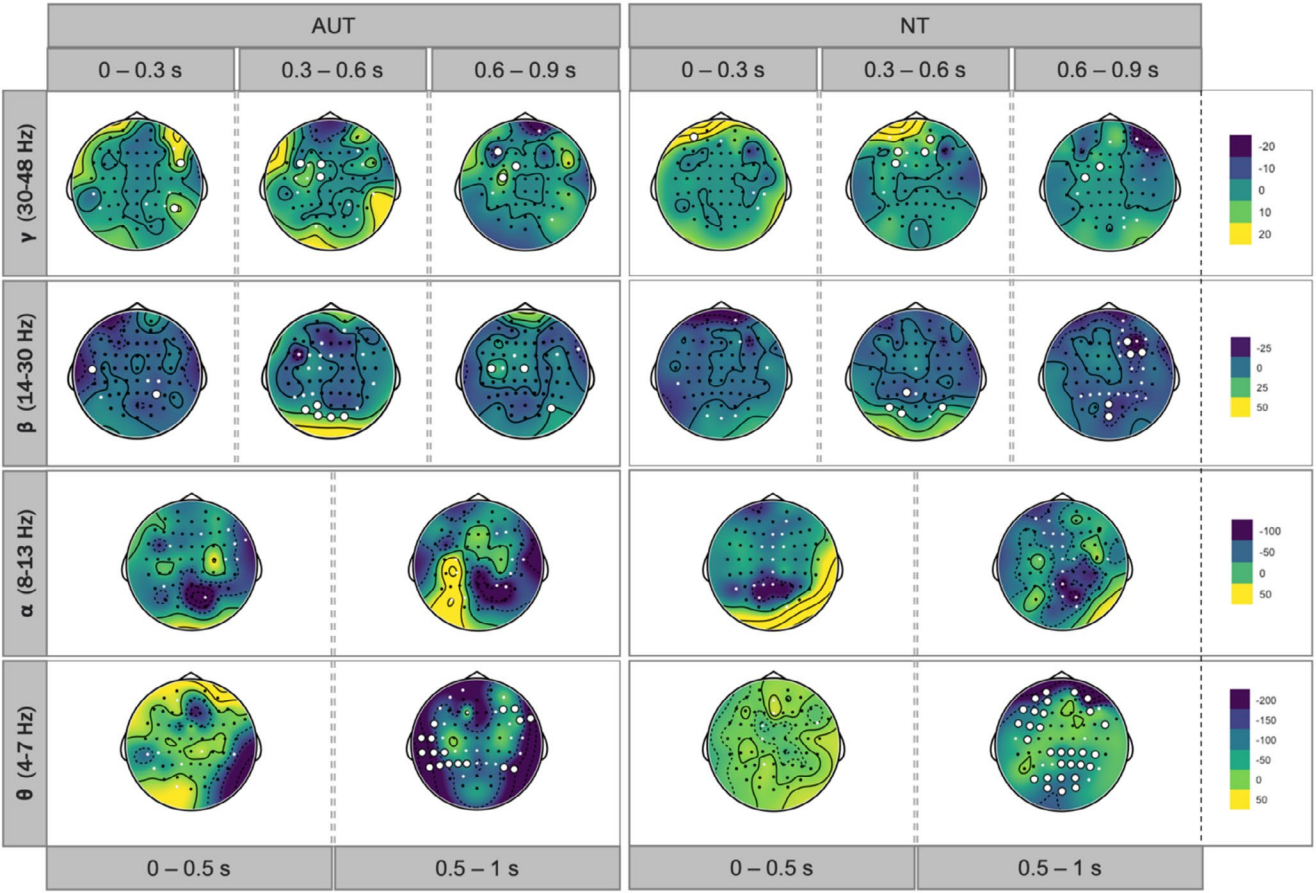
Spatio‐temporal unfolding of MSI across bands. The colors of the topographical maps represent the difference of multisensory (AV) minus the sum of unisensory (A + V) mean power (μV^2^). The circles correspond to the locations of the electrodes, with the large white circles marking the corrected MSI effect at this site and the small white circles representing additional sites with MSI effects, which did not survive the FDR correction.

Figure 5 shows the MSI contrast extended to four frequency bands (theta, alpha, beta, and gamma). Although few points survived FDR correction (note the number of comparisons performed: 61 electrodes × 2 or 3 bins = 122 or 183 comparisons per group and band), these plots offer the spatial distribution of time‐resolved information about multisensory effects in a wide range of frequencies in the brains of autistic and neurotypical adults. For example, these plots reveal that the theta band may be particularly interesting for future explorations in the context of MSI, and particularly importantly for the context of autism—it seems clear that MSI is more widely spread in NT than in AUT in the 0.5–1 s after the onset of the target word. Given that the approach taken here (calculating MSI effects with oscillatory power in complex stimuli) is very novel and there are not many available data for similar insights, we believe that this first overview may serve as a base for other researchers to generate their hypotheses. Thus, we leave it to them to interpret the descriptive results.

### 3.7 Exploratory Analyses: Correlations Between Neural MSI, Behavioral Audio‐Visual Gain, and Individual Traits

In short, there were no significant correlations between the behavioral benefit in accuracy and alpha suppression (*N* = 70), nor between either of these and AQ, IQ, age, LSAS, or ADOS (in autistic participants with ADOS score; *N* = 26). Groups did not differ in their false positive and false negative rates for the catch trials, and sex at birth as a predictor did not improve either the neural or the behavioral benefit models (see Supporting Information S1 for details).

## 4 Discussion

We investigated behavioral and neural correlates of MSI of speech in autism, in a socially relevant complex environment. The key finding is that while both autistic and neurotypical groups benefited behaviorally to a similar extent from congruent multisensory information when performing a speech‐in‐noise comprehension task, they differed in the underlying neural processing mechanisms. As expected, both groups showed greater alpha suppression during audio‐visual speech perception than in either of the unisensory conditions. However, only the neurotypical group showed responses consistent with MSI based on non‐linear interactions between auditory and visual inputs. Instead, the autistic group showed responses consistent with additive audio‐visual processing. This pattern of results suggests that while the autistic and neurotypical brains realize the MSI function differently, both result in efficient behavioral outcomes. This finding (1) extends previous results obtained in simpler, more constrained conditions to more ecologically valid contexts; (2) furthers current MSI research on autistic populations to adulthood, a developmental stage that is potentially informative but underrepresented in this line of research; and (3) considers behavioral and neural integration effects, including group differences between simple additive and interaction‐based MSI processes.

### 4.1 Similar Behavioral Benefits Across Groups

In line with our predictions, autistic participants showed improved speech‐in‐noise comprehension in the audio‐visual (AV) compared to auditory‐only (A) and visual‐only (V) conditions (similar to neurotypicals). This was further supported by faster RTs in AV than in A or V trials. Furthermore, the groups did not significantly differ in the extent of this behavioral benefit. Previous studies showed that autistic children and adolescents benefit less from multimodal speech information than their neurotypical peers (Irwin et al. 2011; Smith and Bennetto 2007; Stevenson et al. 2017; Brandwein et al. 2013; Foxe et al. 2015), these differences wane with age (Beker et al. 2018; Foxe et al. 2015) and may vanish altogether in adults (Magnée et al. 2008). Thus, our data provide evidence to support the age‐related catch‐up process in multisensory gain in autism.

At the same time, it is possible that iconic gestures were particularly beneficial for speech‐in‐noise comprehension in this study—perhaps more than other visual articulators, but this cannot be disentangled within our study design. Indeed, the average accuracy rates for visual‐only trials (89% correct) were higher than for auditory‐only trials (73% correct) in both groups. Note that most audio‐visual speech studies report superior accuracy in the auditory modality compared to the visual (Ross et al. 2007; Sumby and Pollack 1954; Jaekl et al. 2015; for reviews, see Campbell (2008), Navarra et al. (2012)). Interestingly, these studies often exclude gestures or head movements. When this constraint is removed, the modality imbalance may revert to favor the visual channel, at least under certain circumstances. While the role of gestures is established in communication (Kita and Emmorey 2023; McNeill 2000), little is known about how gestures and visual cues like lip movements interact with spoken language in naturalistic settings. This interplay is essential for understanding the general communication dynamics, especially in autism.

The higher accuracy rates observed in the visual‐only trials, compared to other studies with visual speech with or without gestures (Drijvers and Özyürek 2017, 2020), may suggest that gestures in our design provided a particularly strong benefit to word recognition. It also suggests that many of the gestures used in this study were easy for participants to decode. Previous research showed that speech‐gesture integration processes differ between iconic gestures that can be decoded on their own vs. iconic gestures that can be decoded only with accompanying speech (Willems et al. 2009). Thus, it is possible that our findings may not generalize to the latter type of iconic gestures. This could be an important topic for future research. Finally, in our accuracy models, we incorporated random intercepts and slopes for each stimulus, accounting for item‐specific variability. This allowed us to control how different words with accompanying gestures and visual articulators (as these cannot be separated from our stimuli) were perceived across conditions.

Although autism research has focused primarily on gesture production, leaving comprehension understudied (Dimitrova et al. 2017), autistic children understand gesture and speech similarly to their typically developing peers (Dimitrova et al. 2017; Dimitrova and Özçalışkan 2022) and can benefit from watching iconic gestures in terms of focusing on the narrator and recalling the content (Dargue et al. 2021; Kurt 2011). To date, only one other study has investigated the gestural benefit in a speech‐in‐noise task in autistic adults and, similarly to us, found no difference in the size of the gestural enhancement between the groups (Mazzini et al. 2023). We used stimuli rich in visual articulators—lip movements, iconic gestures, and torso/face dynamics—to create a more naturalistic and integrated speech‐processing environment. As such, our study did not aim to isolate the specific gestural enhancement from the overall multisensory benefit in speech processing. Regardless, the clear behavioral advantage of processing multimodal speech is evident in autistic adults and supports the previous (albeit scarce) literature.

Nevertheless, behavioral differences might still emerge under more challenging conditions, with different attentional demands, or under different signal‐to‐noise ratios. For example, one limitation of the current study is the lack of varying noise levels, which would have allowed a direct comparison of performance under different signal‐to‐noise conditions. It is possible that the lack of group differences in word recognition accuracy in our results may be related to ceiling effects in the AV condition (see Figure 2). Because the extent to which humans benefit from visual articulators (including iconic gestures) in degraded speech varies strongly with the amount of noise in the signal (Drijvers and Özyürek 2017), decreasing signal‐to‐noise ratio in our stimuli could potentially reveal group differences. In other words, it is possible that in more difficult listening conditions, autistic and neurotypical individuals show differences in the behavioral benefit from multisensory information.

It is also worth noting that although our design was not optimal for capturing MSI effects in reaction time due to the delayed presentation of response options, autistic and neurotypical participants performed similarly across the three conditions. Even though we found a marginally significant group‐by‐condition interaction, the Bayes Factor provided strong evidence against such an effect, and no pairwise comparisons survived when multiple comparison corrections were applied. However, autistic participants were generally significantly slower in their RTs than the neurotypical group, consistent with findings from multiple studies across different cognitive domains and tasks (Zapparrata et al. 2023; Jertberg et al. 2024). Because RTs may reflect multiple sequential or overlapping processes, the underlying cause of this slower response pattern in autism remains unclear, with possible explanations ranging from slower processing speed to differences in meta‐cognition, motor coordination, or sensory processing.

### 4.2 Different Neural MSI Mechanisms Between the Groups

There are two main findings in our neural data. First, in terms of simple contrasts, we found significantly stronger alpha suppression in AV compared to A and V conditions in both autistic and non‐autistic adults, with no group differences. We interpret the increased alpha suppression here as an indicator of heightened integration load (Matyjek et al. 2024), reflecting the processing demands involved in combining the two information channels. The absence of group differences is consistent with a recent fMRI study using a naturalistic narrative audio‐visual perception task, which found no significant differences in neural activation between autistic and neurotypical participants for bimodal gain (AV − A) (Ross et al. 2024). Second, only the neurotypical group showed a significant MSI *interaction* effect, calculated via the AV vs. A + V contrast. What is more, the size of the MSI effect was significantly larger in the neurotypical than the autistic group.

The AV vs. A + V contrast aims to determine whether the benefit from AV presentation could be accounted for by merely adding auditory and visual neural responses (Giard and Peronnet 1999). Using this approach, two studies measured sensory ERPs to non‐social, low‐level stimuli and found delayed and spatially limited MSI effects in autistic compared to non‐autistic children and adolescents (Stefanou et al. 2020; Brandwein et al. 2013). In autistic children, this was accompanied by lesser behavioral benefit, but there were no behavioral group differences in adolescents (in line with the studies showing age‐related improvements of behavioral MSI in autism; Beker et al. 2018; Foxe et al. 2015). In contrast, two other studies addressed MSI effects in ERPs for social stimuli in adult autistic and non‐autistic participants (Magnée et al. 2008, 2011) and found no group differences in the AV vs. A + V (referred to as “lower‐order MSI”). However, they also investigated “higher‐order MSI”, defined as congruency effects, which were observed in the neurotypical, but not autistic group. This was interpreted as an impairment in higher‐order integration of complex information in autism. Our results contrast the “lower‐order MSI” effect in these studies. Arguably, our stimuli are more *complex*, including full words embedded in semantic, pragmatic, and social context, and multiple visual cues (in contrast to short non‐semantic utterances and non‐speech stimuli). Perhaps this complexity aligns better with the “higher‐order” MSI effects reported by Magnée et al. (2008), Magnée et al. (2011). This suggests that the autistic brain integrates multisensory information similarly to the neurotypical brain when the stimuli are relatively simple. However, with more complex and context‐dependent stimuli, as in our speech task, group differences emerge. Specifically, the autistic brain seems to rely less on the integration between sensory channels and more on additive processing, as reflected by less alpha suppression (i.e., a potential proxy of integration load) in the AV vs. A + V contrast.

Thus, one possible interpretation of our data is that the neurotypical group, showing greater alpha suppression in the AV vs. A + V contrast, integrates the two channels regardless of stimulus complexity. In contrast, the autistic group might use additive computations when faced with the complex input used here, despite being capable of integration for simpler stimuli. This shift could indicate that MSI effects are less automatic in autism, making additive computations more cost‐efficient when processing demands are high. Because we did not manipulate stimulus complexity directly, we can only speculate—based on previous studies (Magnée et al. 2008, 2011)—that complexity is a defining factor. This interpretation represents a hypothesis generated by our study, which should be tested in future research.

Regardless, our results extend previous findings to more naturalistic stimuli and contribute to the literature on neural MSI atypicalities in autism. Importantly, these atypicalities should not be interpreted as “impairments,” as there were no group differences in the behavioral benefits from multisensory information. When two brains achieve the same behavioral outcomes through different mechanisms, these variations are differences, not impairments.

### 4.3 Mismatch Between Behavioral and Neural MSI Correlates

One feasible explanation for the mismatch between behavioral and neural MSI correlates is that autistic adults, unlike children, have developed compensatory strategies for integrating multisensory information, leading to no behavioral differences (at least those measured in this study) in adulthood. This aligns with reports of a developmental delay in MSI in autistic children, estimated to be around 6 years (Foxe et al. 2015). The specific nature of these compensatory mechanisms remains unclear.

On the one hand, higher‐order attentional processes in autistic adolescents may activate MSI at later stages, compensating for reduced early integration compared to neurotypicals (Stefanou et al. 2020). Although we cannot directly test this with our data, our exploratory analysis indicates that theta band activity might reveal significant MSI processes, with neurotypicals showing more integration effects than autistics, particularly in the second time window (0.5–1 s post‐stimulus). This supports the later‐stage compensatory processing in autism.

On the other hand, autistic individuals may need active attention to initiate early MSI (Russo et al. 2010; Brandwein et al. 2013; Magnée et al. 2008; Dunn et al. 2008; Whitehouse and Bishop 2008). For example, an early MSI effect was observed only in neurotypicals in a passive task (Russo et al. 2010), but both groups showed early MSI in an active task with explicit attentional demands (Brandwein et al. 2013). Also, manipulating attention revealed typical brain responses in autism during low‐level MSI tasks and selective attention, but not during divided attention (Magnée et al. 2011). Thus, while MSI depends on attentional demands (Morís Fernández et al. 2015; Alsius et al. 2005, 2007, 2014; Tiippana et al. 2004), autistic individuals might rely more on selective attention, which could manifest similarly to MSI impairments (Marco et al. 2011; Magnée et al. 2008). In our study, participants were required to divide attention between audio and visual channels by detecting random targets, with no significant differences in false alarm or miss rates between groups. However, we cannot be certain that this ensured equal divided attention between the groups. Future studies should explicitly manipulate attention for precise calibration.

Finally, attention engagement might differ for social stimuli in autism. Autistic adults may perform worse with background speech noise than neurotypicals (Alcántara et al. 2004), although this does not replicate in autistic traits (Tsuji and Imaizumi 2024). We used pink noise instead of socially related noise to degrade speech comprehension, and further research is needed to address social vs. non‐social noise in naturalistic stimuli in autism. Attention engagement may also depend on the complexity of the stimuli. Specifically, highly complex stimuli—such as speech with multiple visible articulators embedded in a socio‐pragmatic context—might become sensory‐wise overwhelming for autistic individuals. When the information load imposes high demands on attention, integration is less likely, and the most efficient strategy may be to revert to cost‐efficient, additive processes. While this is merely a speculation in light of the results of this study, it presents an interesting avenue for future research.

### 4.4 Clinical Implications

The findings from this study have significant clinical implications, particularly for interventions aimed at enhancing social communication in autistic adults. As MSI difficulties may underlie higher‐order socio‐communicative issues, multisensory processing is a promising target of intervention in autism (Cascio et al. 2016; Kawakami and Otsuka 2021). Indeed, several multisensory interventions have already been proven beneficial for the development of MSI and other basic functions in infants and children (Kawakami and Otsuka 2021). Here, we introduce adult data and show that despite differing neural mechanisms of MSI, both autistic and neurotypical adults achieved comparable behavioral outcomes (at least in the accuracy of word recognition tested here). This suggests that interventions should focus on supporting compensatory mechanisms that autistic individuals naturally develop with time. Moreover, accumulating evidence, including our findings, suggests that both the production and perception of gestures significantly enhance communicative performance in both autistic and neurotypical individuals (e.g., Özçalışkan et al. 2017). Thus, clinically, incorporating rich, naturalistic audio‐visual stimuli, such as gestures alongside speech, may improve communication outcomes in real‐life settings.

### 4.5 Conclusions

Our results show that while autistic adults benefit behaviorally from multisensory speech information, they do not show the same neural MSI as neurotypicals. Despite these neural differences, both groups achieved comparable behavioral outcomes, suggesting that the variations in neural mechanisms do not imply impairments. Attention may play a critical role, with autistics possibly relying on compensatory strategies developed over time.

## Author Contributions

Conceptualization: M.M., S.K., and S.S.F. Data collection and curation: M.M. Analysis: M.M. and M.T.C. Visualization: M.M. Funding acquisition: M.M. and S.S.F. Original draft preparation: M.M. Reviews: All authors. Supervision: S.K. and S.S.F.

## Ethics Statement

The study protocol and data handling were approved by the Institutional Committee for Ethical Review of Projects at University Pompeu Fabra (CIREP‐UPF; number 258).

## Consent

All participants gave written consent to participate in the study.

## Conflicts of Interest

The authors declare no conflicts of interest.

## Supporting information


**Data S1.** Supporting Information.

## Data Availability

The data and analysis code will be made publicly available upon publication, at https://osf.io/2b6s3/ (subproject “MSI in autistic vs. neurotypical adults”).

## Endnotes

^1^
The term “neurotypical” refers to individuals with typical neurological development and functioning, but it often refers to individuals who are simply not autistic. This overlooks diversity within non‐autistic populations. Nevertheless, in autism research, comparison groups are usually controlled for neurodivergent traits (e.g., anxiety) or clinical conditions (e.g., schizophrenia). Therefore, we use “neurotypical” interchangeably with “non‐autistic” in this context.

^2^
We use identity‐first language (autistic person) in this paper, as it is preferred by the majority of English‐speaking individuals on the autism spectrum (Kenny et al. 2016), reduces stigma (Gernsbacher 2017), and aligns with the preferences of all members of the autistic community directly or indirectly involved in this work.
