# Supplementary material for: Multisensory Integration of Naturalistic Speech and Gestures in Autistic Adults
Source: Autism Res. 2025 Apr 17;18(6):1156–69. doi: 10.1002/aur.70042 (PMC12166514; doi:10.1002/aur.70042)
Supplement: Supplementary file 1 — Data S1. Supporting Information. [file AUR-18-1156-s001.docx]

Supplementary material to:

**Multisensory Integration of Naturalistic Speech and Gestures in Autistic Adults**

Matyjek, Kita, Torralba Cuello, Soto Faraco

Table of Contents

[1. Additional diagnostic information for AUT 2](#_Toc182478976)

[2. Artifact-free segments in EEG data 4](#_Toc182478977)

[3. Accuracy in both groups 4](#_Toc182478978)

[4. Behavioural benefit across groups – maximum unisensory approach 5](#_Toc182478979)

[5. Reaction times in both groups 5](#_Toc182478980)

[6. Simple bimodal benefit and interaction MSI effects in alpha suppression for both groups 7](#_Toc182478981)

[7. Secondary analysis 7](#_Toc182478982)

[8. Secondary analysis: responses to targets in catch trials 9](#_Toc182478983)

# Additional diagnostic information for AUT

| **Parti-cipant** | **Diag-nosis** | **Year of diag-nosis** | **ADHD** | **Depre-ssion** | **Anxiety** | **Other diag.** | **Medication** | **Medication reason** | **ADOS-2 used** | **ADOS-2 total score** | **ADOS-2 comm. score** | **ADOS-2 social score** | **ADI-R used** |
| --- | --- | --- | --- | --- | --- | --- | --- | --- | --- | --- | --- | --- | --- |
| 1 | ASD, I | 2019 | No | No | Yes | ADD | Decentan | mood regulator | Yes | 30 | - | - | No |
| 2 | ASD, I | 2021 | No | Yes | Yes | - | Paroxetina, Trazadana | depression | Yes | 9 | 4 | 4 | No |
| 3 | ASD, I | 2022 | No | No | Yes | - | Gabapetina | anxiety | Yes | 8 | 2 | 6 | Yes |
| 4 | ASD, I | 2016 | No | Yes | Yes | - | Escifalopram, Lorazepan | depression, anxiety | No | - | - | - | Yes |
| 5 | ASD, I | 2022 | No | Yes | Yes | - | - | - | Yes | 6 | 2 | 4 | No |
| 6 | ASD, I | 2013 | No | Yes | No | - | Citalopram | depression | No | - | - | - | No |
| 7 | ASD, I | 2022 | Yes | Yes | Yes | - | Concerta | ADHD | Yes | 7 | 2 | 5 | No |
| 8 | ASD, I | 2015 | No | Yes | No | - | Fluoxetina | depression | No | - | - | - | No |
| 9 | ASD, I | 2015 | Yes | No | Yes | PTSD | Brintellix, Lorazepam | depression, anxiety | Yes | 13 | 5 | 8 | No |
| 10 | ASD, I | 2021 | No | Yes | Yes | - | Diazepam | anxiety | Yes | 10 | 4 | 6 | Yes |
| 11 | ASD, I | 2023 | Yes | No | No | - | Concerta | ADHD | Yes | 13 | 5 | 8 | No |
| 12 | ASD, I | 2020 | No | No | No | ADD | Elvanse | ADD | Yes | 7 | 3 | 4 | No |
| 13 | ASD, I | 2021 | No | Yes | No | - | Escifalopram | depression | Yes | 10 | 6 | 4 | No |
| 14 | ASD, I | 2023 | No | Yes | Yes | - | - | - | Yes | 8 | 2 | 6 | No |
| 15 | ASD, I | 2022 | No | Yes | Yes | migraine | Fluoxetina | depression | Yes | 11 | 5 | 6 | No |
| 16 | ASD, I | 2017 | Yes | No | No | OCD | - | - | Yes | 7 | 2 | 5 | Yes |
| 17 | ASD, I | 2020 | No | Yes | Yes | - | Lorazepam, Paroxetina | depression, anxiety | Yes | 9 | 2 | 7 | Yes |
| 18 | ASD, I | 2022 | Yes | No | No | - | Elvanse | ADHD | Yes | 2 | 2 | 0 | No |
| 19 | ASD, I | 2023 | No | Yes | No | - | Sertralina | depression | Yes | 16 | 6 | 10 | No |
| 20 | ASD, I | 2019 | No | No | No | - | Carbamazepina | convulsions | Yes | 12 | 3 | 9 | Yes |
| 21 | ASD, I | 2022 | No | No | No | - | - | - | Yes | 7 | 2 | 5 | Yes |
| 22 | ASD, I | 2017 | No | Yes | Yes | - | - | - | Yes | 8 | 2 | 6 | No |
| 23 | ASD, I | 2021 | No | No | No | - | - | - | Yes | 9 | 4 | 5 | Yes |
| 24 | ASD, I | 2018 | No | No | No | - | - | - | Yes | 7 | 3 | 4 | No |
| 25 | ASD, I | 2022 | No | No | No | - | - | - | Yes | 15 | 5 | 10 | Yes |
| 26 | ASD, I | 2022 | No | Yes | Yes | - | Heipram | anxiety | No | - | - | - | Yes |
| 27 | ASD, I & II | 2023 | Yes | No | Yes | PTSD | - | - | Yes | 12 | 2 | 10 | Yes |
| 28 | ASD, I | 2023 | No | Yes | Yes | - | Fluoxetina | anxiety | Yes | 11 | 4 | 7 | Yes |
| 29 | ASD, I | 2023 | No | No | Yes | OCD | Citalopram | anxiety | No | - | - | - | No |
| 30 | ASD, I | 2022 | Yes | Yes | Yes | - | Concerta, Ribotril | ADHD, anxiety | No | - | - | - | Yes |
| 31 | ASD, I | 2020 | No | No | No | panic attacks | Risperdal, Noiafren | panic | No | - | - | - | No |
| 32 | ASD, I | 2023 | No | No | No | ADD, OCD | Sertralina | depression | No | - | - | - | No |
| 33 | Asperger's | 2008 | 1 | 0 | 0 | epilepsy | Vimpat (epilepsy), Strattera (ADHD), Sertralina (depression), Depakine (epilepsy), Quetiapina (schizophrenia), Selincro (alcohol) | epilepsy, ADHD, depression, schizophrenia, alcohol | No | - | - | - | No |
| 34 | TEA, I | 2022 | 0 | 0 | 1 | - | - | - | Yes | 10 | 3 | 7 | Yes |
| 35 | TEA, I | 2020 | 0 | 0 | 0 | - | - | - | Yes | 10 | 3 | 7 | No |

# Artifact-free segments in EEG data

The number of artifact-free segments did not significantly differ across experimental conditions, *F*(2,13.40)=0.81, *p*=.45, and group*condition interaction was not significant, *F*(2,6.30)=0.38, *p*=.68, but there was on average one segment more in NT than in AUT, *F*(1,69.72)=8.42, *p*=.004 (the mean number of segments left for groups and conditions were: AV_AUT_=58.17, A_AUT_=58.26, V_AUT_=58.37, AV_NT_=58.86, A_NT_=59.51, V_NT_=59.89).Further, we calculated the signal-to-noise ration (SNR) on the artifact-free segments (averaged across all electrodes except HEOG and VEOG) with the Brain Vision Analyser's SNR function, i.e., by dividing the average signal power by the average noise power, where noise is calculated as the total of the squares of the differences between the EEG value and the average value, divided by the number of points minus 1, and signal power is the difference between the total power and the noise. The SNRs did not differ between groups, *F*(1,2)=0.09, *p*=.78, or conditions, *F*(2,2)=6.09, *p*=.14.

# Accuracy in both groups

Average accuracy rates for groups and conditions are shown in Supp. Table 1. First, a logistic regression model with condition (AV, A, V) and group (AUT, NT) was built as follows:

glmer(formula = correct ~ cond * group + (1 + cond | ID) + (1 +

cond | verb), data = DF, family = binomial(link = "logit"),

control = glmerControl(optimizer = "bobyqa", optCtrl = list(maxfun = 200000)))

To estimate the global effects of its terms, we compared this model against models with the corresponding terms dropped using a likelihood test. This procedure revealed a statistically significant main effect of condition, *X^2^*(2) = 127.61, *p* < .001, so that accuracy in AV was larger than in A and larger than in V. The odds of responding correctly in the A and V conditions were, respectively, 94% (1-0.06, 95% CI [.04 .10]) and 75% (1-0.25, 95% CI [.17 .40]) significantly (both *p*s < .001) smaller than in the AV condition. Interaction and group effects were not statistically significant (respectively, *X^2^*(2) = 3.40, *p* = .18 and *X^2^*(2) = 1.61, *p* = .20).

Thus, both groups showed the behavioural MSI effect in the accuracy rates of word detection, so that participants were more accurate in bimodal than in unimodal trials. The groups did not significantly differ in their responses in general or for different conditions.

Supp. Table 1: Average accuracy rates in word detection by group and condition. SDs are reported in brackets.

| **Group** | **AV** | **A** | **V** |
| --- | --- | --- | --- |
| **AUT** | 96.53% (3.55) | 72.11% (8.56) | 86.84% (9.13) |
| **NT** | 97.88% (1.77) | 73.95% (7.84) | 90.42% (5.9) |
| **All** | 97.21% (2.87) | 73.03% (8.2) | 88.63% (7.84) |

# Behavioural benefit across groups – maximum unisensory approach

We pre-registered that for each participant we would calculate the behavioural MSI benefit with the following equation: 𝐴𝑉 − max(𝐴, 𝑉), where AV is the percent of correct responses in AV trials, and max(A,V) is the highest percent of correct responses between A and V trials. However, this approach is not optimal, as although it implies that information is being used from both sensory modalities, the information does not necessarily have to be *integrated* ^1^. Thus, in the article we report an alternative measure (probability summation). Nevertheless, here we report the pre-registered analysis for full transparency.

We compared the behavioural benefit (estimated as the difference in accuracy between AV and the largest of A or V) between AUT (mean=9.55) and NT (mean=7.37) with a t-test, which yielded no significant effect, *t*(60.27)=1.38, *p*=.17, (see Supp. Fig. 1). We additionally compared a model including the group factor against an intercept-only model, which yielded BF01=3.19, suggesting weak evidence in favour of the intercept-only model. This result parallels the one reported in the main text with the more conservative approach.


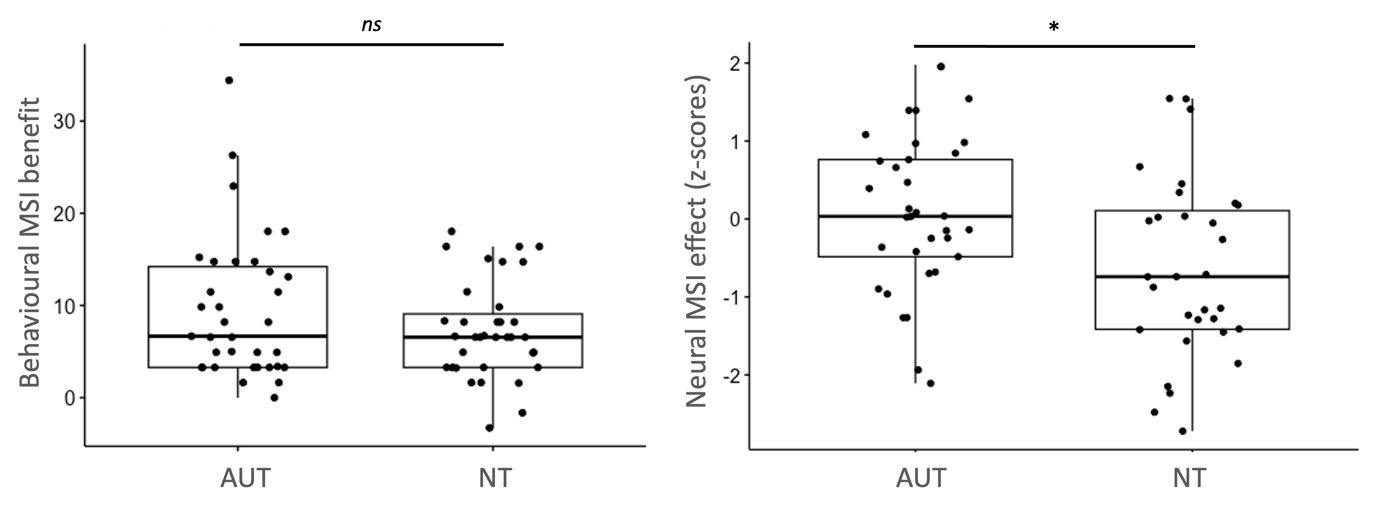


Supp. Fig. 1 Group effects in the behavioural benefit in word recognition calculated with the maximum unisensory equation.

# Reaction times in both groups

Mean RTs per group and condition are shown in Supp. Table 2.

Supp. Table 2: Average reaction times by group and condition. SDs are reported in brackets.

| **Group** | **AV** | **A** | **V** |
| --- | --- | --- | --- |
| **AUT** | 1.18s (0.43) | 1.52s (0.59) | 1.34s (0.53) |
| **NT** | 1.07s (0.32) | 1.38s (0.46) | 1.16s (0.4) |
| **All** | 1.12s (0.38) | 1.45s (0.53) | 1.25s (0.48) |

To analyse RTs, which do not follow normal distribution, we first built two models with Gamma distribution and Inverse Gaussian distribution, and the same fixed and random structure:

1. glmer(RT ~ cond*group + (1|ID) + (1|verb), data = RT, family=Gamma(link="identity"))
2. glmer(RT ~ cond*group + (1|ID) + (1|verb), data = RT, family=inverse.gaussian(link="identity"))

Then, we compared models 1 and 2 for AIC and BIC, which were both lower for model 1 (AIC_1_ = 9398.974, AIC_2_ = 9556.402, BIC_1_ = 9465.208, BIC_2_ = 9622.636). This model yielded main effects of condition, *X^2^*(2)=1084.21, *p*<.001 and group *X*^2^(1)=5.51, *p*=.02, and their interaction, *X*^2^(2)=6.48, *p*=.04. However, no pairwise comparisons of RTs between the groups survived correction for multiple comparisons (Supp. Table 3). Additionally, the BF01 was 454.43, suggesting strong evidence in favour of a model without the interaction effect.

Together, RTs were faster for AV than V and for V than A, and NT showed faster RTs than AUT, but the groups responded similarly across conditions (Supp. Fig. 2). The distribution of observed RTs in AV trials never surpassed the race model with RTs in A and V trials in either group (Supp. Fig. 3).


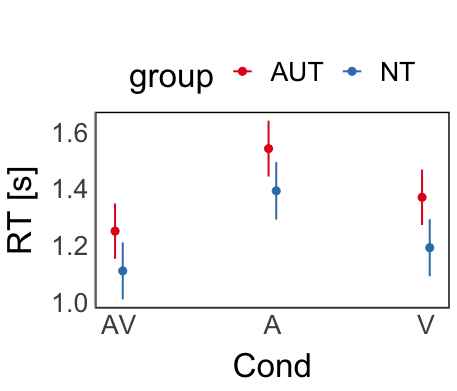


**Supp. Fig. 2: Predicted reaction times for groups and conditions.** Error bars mark 95% CI.


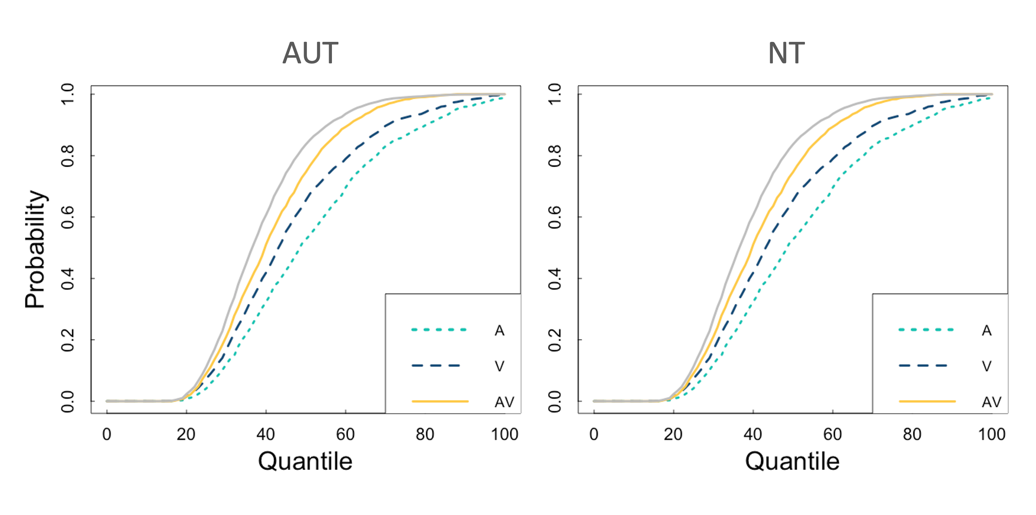


Supp. Fig. 3: Representation of the race model test for each group.

Supp. Table 3: Pairwise comparisons for the effect of condition and group interaction on RTs (with Holm correction).

| **Contrast** | **Est.** | **Std. Error** | **z value** | **p_corr_** |
| --- | --- | --- | --- | --- |
| **AUT - NT** | | | | |
| NT_A_ - AUT_A_ | -0.149 | 0.067 | -2.216 | 0.163 |
| NT_AV_ - AUT_AV_ | -0.140 | 0.067 | -2.107 | 0.206 |
| NT_V_ - AUT_V_ | -0.178 | 0.067 | -2.664 | 0.052 |
| **AUT cond** | | | | |
| AUT_A_ - AUT_V_ | 0.172 | 0.013 | 12.852 | 0.000 |
| AUT_AV_ - AUT_A_ | -0.291 | 0.013 | -22.851 | 0.000 |
| AUT_AV_ - AUT_V_ | -0.119 | 0.011 | -10.725 | 0.000 |
| **NT cond** | | | | |
| NT_A_ - NT_V_ | 0.201 | 0.012 | 16.285 | 0.000 |
| NT_AV_ - NT_A_ | -0.283 | 0.012 | -23.738 | 0.000 |
| NT_AV_ - NT_V_ | -0.082 | 0.010 | -8.060 | 0.000 |

# Simple bimodal benefit and interaction MSI effects in alpha suppression for both groups

**Bimodal benefit in both groups**

We tested the bimodal benefit (AV vs. A and AV vs. V) in both groups by building a linear regression mixed model with condition (AV, A, V), group (AUT, NT), and their interaction as predictors of alpha suppression (with random intercepts for participants). This analysis yielded a main effect of condition, *F*(2,12271.1)=20.71, *p*<.001, with alpha more suppressed in AV than A (*est.*=106.60, *p_corr_*<.001) and in AV than V (*est.*=79.38, *p_corr_*<.001). There were no statistically significant effects of group, *F*(1,70.3)=0.51, *p*=.48, BF01 = 86.01, or condition*group interaction, *F*(2,12271.1)=0.62, *p*=.54, BF01 = 666.72.

**Interaction MSI in both groups**

Contrary to our prediction 3, we found no evidence for interaction MSI effects in AUT, tested with a one-sided t-test of z-scores against 0, *t*(32) = 0.29, *p*=.61. As pre-registered, these z-scores were free of outliers (i.e., values over or below the median +/-2x median absolute deviation). The same test on all the z-scores (including outliers) was also insignificant, *t*(34) = -0.45, *p*=.33. The same test for NT showed a significant result on z-scores without the outliers, *t*(30) = -3.20, *p* = 0.002 (but not when including the outliers, t(34) = -1.31, *p* = 0.1).

# Secondary analysis

We additionally explored possible correlations between our main behavioural and neural outcomes: the behavioural benefit from multisensory information (both for the maximum unisensory (AV – max(A,V) and for the probability summation (AV – (A+V-A*V))), and the z-score for the AV – (A+V) contrast in alpha suppression. All correlations are shown in

Supp. Fig. 4. The neural MSI outcome did not correlate with either of the behavioural benefits. Among all the additional measures (AQ, IQ, age, LSAS, and ADOS - taking only the autistic participants who had an ADOS score; N=26), the only significant correlation was between the behavioural benefit calculated with the probability summation equation and the IQ so that the higher the IQ, the smaller the benefit; this was true for the both groups considered together, but not within either of the groups (see

Supp. Fig. 4 panel A). After removing the z-score outliers (see

Supp. Fig. 4 panel B), the correlation between the PS behavioural benefit and IQ remained significant, and additionally the neural MSI correlated positively with AQ and LSAS (all only for the two groups together but not withing groups). We stress that these are exploratory correlations on a small number of data points, with no corrections for multiple comparisons. Thus, we consider them not significant in the light of this study.

Finally, we checked if the neural benefit can be predicted with the behavioural benefit (calculated either with maximum unisensory or probability summation approach), age, AQ, LSAS, and biological sex in a regression model. We also build a model predicting the behavioural benefit with the neural benefit and the remaining secondary predictors. In either model, none of the predictors were statistically significant.


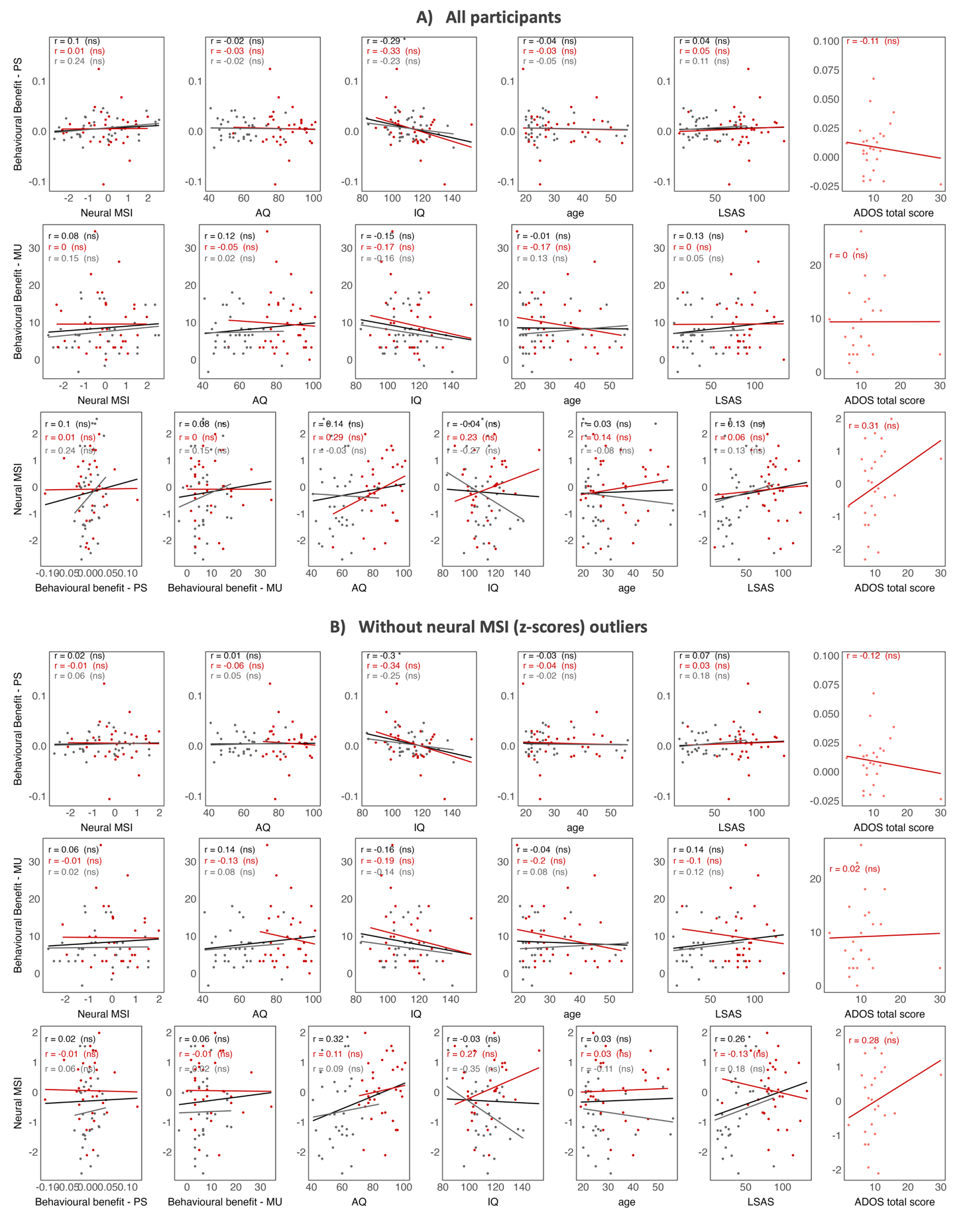


Supp. Fig. 4 Exploratory correlations between the behavioural and neural MSI outcomes and AQ, IQ, age, LSAS, and ADOS (for AUT participants only). Red, grey and black dots/lines mark data points and regression line for, respectively, AUT, NT, and both groups together. PS = probability summation; MU = maximum unisensory.

# Secondary analysis: responses to targets in catch trials

To ensure that participants paid attention to the task in both sensory channels, participants were asked to monitor for occasional glitches (catch trials) in either the visual or the auditory channel (frozen frames or a burst of noise in the microphone, respectively). There were 9 catch trials in the auditory and 10 in visual modality. The mean false positive and false negative responses per group are shown in Supp. Table 4.

Supp. Table 4 Mean false positive and false negative responses per group. FP = false positive, FN = false negative. For FP, AV/A/V signify the condition of the trial. For FN, A/V signify the modality in which the target was presented. SDs are reported in brackets.

| **Group** | **FP in AV** | **FP in A** | **FP in V** | **FN in A** | **FP in V** | **FP** | **FN** |
| --- | --- | --- | --- | --- | --- | --- | --- |
| **AUT** | 0.43  (0.9) | 2.57  (10.10) | 3.06  (6.61) | 0.89  (1.4) | 0.89 (1.105) | 6.06  (16.72) | 1.77  (1.78) |
| **NT** | 0.14  (0.5) | 1.89  (6.11) | 0.31  (0.80) | 0.94  (1.37) | 0.40  (0.65) | 2.34  (6.11) | 1.34  (1.51) |
| **All** | 0.29  (0.73) | 2.23  (8.30) | 1.69  (4.87) | 0.91  (1.39) | 0.64  (0.93) | 4.2  (12.64) | 1.56  (1.66) |

Some autistic participants (N=5) and one neurotypical participant exhibited particularly high (>10 across conditions) false positive rates (i.e., reporting a target when there was none; see the code/html file with all results), which was due to not understandings of the instructions specific to the targets (even after passing the training). After receiving additional explanations from the experimenter during the breaks between blocks, these participants significantly reduced their false positives. Regardless of including or excluding these participants, there were no significant differences in false positive, of false negative rates between the groups (for all participants, FP: *t*(42.93) = 1.23, *p* = .22, FN: *t*(66.25) = 1.08, *p* = .28).
